# Bumble Bees (*Bombus terrestris*) Use Time-Memory to Associate Reward with Color and Time of Day

**DOI:** 10.3390/insects14080707

**Published:** 2023-08-14

**Authors:** Ozlem Gonulkirmaz-Cancalar, Oded Shertzer, Guy Bloch

**Affiliations:** 1 Department of Ecology, Evolution, and Behavior, The Alexander A. Silberman Institute of Life Sciences, The Hebrew University of Jerusalem, Givat-Ram, Jerusalem 91904, Israel; ozlem.gonulkirmaz@mail.huji.ac.il (O.G.-C.); oded.shertzer@mail.huji.ac.il (O.S.); 2 The Federmann Center for the Study of Rationality, The Hebrew University of Jerusalem, Jerusalem 91904, Israel

**Keywords:** circadian rhythm, time-memory, bumble bees, learning, foraging

## Abstract

**Simple Summary:**

Honey bees are famous for their capacity to precisely time their visits to flowers to maximize food reward, but it is not known whether similar “time-memory” exists in other bees that forage over shorter distances from their nests. Here, we tested whether bumble bees that live in smaller colonies can associate reward with time of day and color. We trained bumble bee workers to feed on yellow or blue artificial flowers during either the morning or evening, respectively. During the test day, we presented non-rewarding flowers and recorded the behavior of the foraging bees. We found that trained bees preferred yellow flowers during the time corresponding to the morning training time and blue flowers during the time corresponding to the evening training time. These observations show that bumble bees can associate time of day with a specific color and reward, suggesting that time-memory is not limited to species such as honey bees that forage over long distances and time periods.

**Abstract:**

Circadian clocks regulate ecologically important complex behaviors in honey bees, but it is not clear whether similar capacities exist in other species of bees. One key behavior influenced by circadian clocks is time-memory, which enables foraging bees to precisely time flower visitation to periods of maximal pollen or nectar availability and reduces the costs of visiting a non-rewarding flower patch. Bumble bees live in smaller societies and typically forage over shorter distances than honey bees, and it is therefore not clear whether they can similarly associate reward with time of day. We trained individually marked bumble bee (*Bombus terrestris*) workers to forage for sugar syrup in a flight cage with yellow or blue feeders rewarding either during the morning or evening. After training for over two weeks, we recorded all visitations to colored feeders filled with only water. We performed two experiments, each with a different colony. We found that bees tended to show higher foraging activity during the morning and evening training sessions compared to other times during the day. During the test day, the trained bees were more likely to visit the rewarding rather than the non-rewarding colored feeders at the same time of day during the test sessions, indicating that they associated time of day and color with the sugar syrup reward. These observations lend credence to the hypothesis that bumble bees have efficient time-memory, indicating that this complex behavior is not limited to honey bees that evolved sophisticated social foraging behaviors over large distances.

## 1. Introduction

Endogenous rhythms of about a day (circadian) are ubiquitous in plants, fungi, and animals. It is commonly accepted that these rhythms are functionally significant because they allow organisms to coordinate their physiology and behavior with the 24-hour rotation of planet Earth around its axis [1,2,3,4].

Many behaviors are influenced by circadian clocks. For example, in the best-studied insect, *Drosophila melanogaster*, it has been shown that behaviors such as sleep, locomotor activity, learning, eclosion, feeding, courtship, mating, egg laying, and time-memory are influenced by circadian pacemakers [5,6,7,8,9]. In honey bees, another important insect model [8], the circadian clock influences complex behaviors such as sun compass orientation, timing visits to flowers, dance communication, division of labor among worker bees, social coordination of activity, and queen egg laying [10,11,12]. For example, honey bees rely on their circadian clock to compensate for the sun’s movement during their “waggle dance,” which conveys azimuth information by referring to the location of the sun. This sophisticated communication system allows honey bees to recruit nestmates to flowers up to 10 km away from their nest [13,14].

Here, we focus on time-memory that has been explored in diverse, yet limited in number, animal species such as birds, flies, ants, fish, and mice [15,16,17,18,19]. Already at the beginning of the twentieth century, it was observed that honey bees show daily foraging rhythms. Later time-training experiments established that they can associate time of day with nectar (or sugar syrup) reward at a specific location [20,21]. This complex behavior is commonly termed time-memory, time-sense, or Zeitgedächtnis (the original name coined in German). Later studies extended this finding to show that honey bees can learn up to nine different times during the day or time visits to different locations (reviewed in [11]). Time-memory is assumed to be functionally significant because it improves foraging efficiency and decreases energy expenditure and predation risk associated with visiting flower patches during times with low or no reward. Although it is now well established that the Western honey bee (*Apis mellifera*) uses sophisticated time-memory, it is yet unknown to what extent this capacity is generalized to other species of bees.

Here, we asked whether bumble bees use similar time-memory to optimize their foraging activity. Bumble bees are phylogenetically related to honey bees, and many species are important pollinators of agricultural crops and natural ecosystems [22]. We studied *Bombus terrestris*, which, by contrast to honey bees, live in annual colonies that are about two orders of magnitudes smaller in population size (several hundred compared to several tens of thousands of bees per colony, respectively [23]). Consistent with their annual life cycle and small colonies, bumble bees typically forage in their nest vicinity and rarely exceed a distance of 800 m [24,25]. Thus, the cost of mistakenly visiting flowers at a time of low or no reward can be assumed lower than that for long-distance-foraging honey bees. By contrast to honey bees, which evolved a sophisticated scouting and recruiting system that leads to temporal-spatial foraging groups, there is no evidence that bumble bees can communicate directions to floral resources in a way comparable to honey bee dance communication [26,27,28]. To the best of our knowledge, there is also no conclusive empirical evidence that bumble bees use their circadian clock for time-compensated sun compass orientation. 

Given that time-memory has the potential to improve foraging efficiency, we hypothesized that *B. terrestris* has time-memory capacity. To test this hypothesis, we trained bumble bee foragers in a flight cage to associate yellow or blue artificial flowers with the time of sugar syrup reward, and we tested their ability to visit the flowers at the same time of day but on a test day with no reward. In Experiment 2, in which we had a larger sample size, we also started to assess the influence of body size and age/experience on time learning.

## 2. Materials and Methods

### 2.1. Bees

We conducted two experiments, each with a different colony, during September 2020 and July 2021 at the Bee Research Facility of the Hebrew University of Jerusalem, Givat Ram, Israel. Bumble bee colonies were purchased from Bio-Bee Biological Systems Ltd., Kibbutz Sde-Eliyahu, Israel. We received the colonies approximately 2–4 days after the emergence of the first worker. Each colony contained a queen, 5–10 workers, and brood at various stages of development. We transferred the colonies into wooden nest boxes (30 × 23 × 20 cm) with transparent Plexiglass covers. The colonies were provisioned with ad libitum pollen paste (pollen collected by honey bees mixed with commercial sugar syrup from Bio-Bee Biological Systems Ltd., Kibbutz Sde-Eliyahu, Israel). We kept the colonies under constant darkness in an environmental chamber (27–29 °C; 40–60% relative humidity [RH]). We used dim red lighting during feeding, inspection, or when observing the colonies.

### 2.2. Time Training

**Figure 1:**
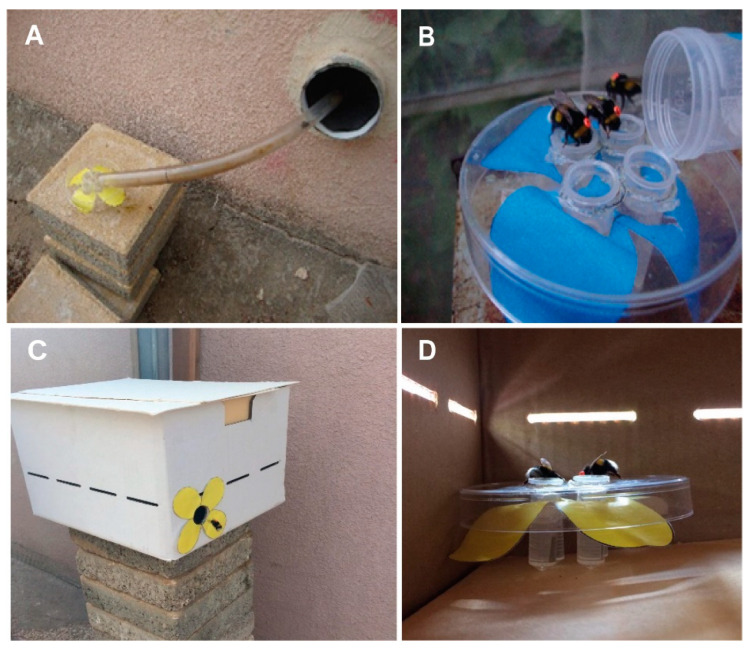
Methodological details for the first experiment testing time-memory in *Bombus terrestris*. (**A**) The tube connecting the colony to the flight cage with an artificial yellow flower at its distant end. (**B**) A four-tube feeder on top of a blue artificial flower that was filled with sugar syrup on training days and with only water on the test day. Three tagged workers are seen feeding from the feeder. (**C**) A flower box in the flight cage. A trained worker is seen flying into the opening. (**D**) A look inside a flower box. Bees are seen feeding on the artificial yellow flower inside the box.

We began the training sessions when the colonies contained approximately 50 workers. We added commercial sugar syrup to 3–4 nectar pots placed in the colony right after the evening session in order to avoid food shortage during the night. To start the training, we connected the colonies to an external flight cage with a transparent PVC tube (Figure 1A).

**Experiment 1.** We connected two colonies of similar age and size, containing approximately 50 workers, to the outside with transparent PVC tubes. The tube fitted to the first colony (Control) was opened outside, allowing the bees to forage freely in the area around the Bee Research Facility. The second colony was connected to an external flight cage (L 510 × W 210 × H 215 cm) in which they foraged on artificial flowers (Figure 1A). We prepared the artificial flowers with either yellow or blue color, equipped with 2 mL Eppendorf centrifuge tubes into which we provisioned commercial sugar syrup (from Bio-Bee Biological Systems Ltd., Kibbutz Sde-Eliyahu, Israel). We washed the flowers, including the sugar syrup feeder, with soap and 70% ethanol at the end of every day to remove any scent marks that may have been deposited by visiting bees [29,30,31]. The flower shape with four petals was printed on paper using a standard office printer and was attached under a Petri dish cover (90 mm in diameter) (Figure 1A,B,D). 

Foraging bees were allowed to explore the flight cage during the first five days of the training period. The sugar syrup supply inside the nest was removed at the end of this period, motivating the bees to search for food outside. On Day 6 (start of training), we presented the artificial flowers filled with commercial sugar syrup during the day without limiting the time of the reward. We first placed the flowers right at the nest entrance (Figure 1B) and confirmed that foragers drank syrup from the artificial flowers. Starting on Day 7, we gradually (~ every hour) moved the artificial flowers farther away from the nest entrance, until they reached two boxes (40 × 30 × 20 cm) placed in the far corners of the flight cage, about 470 cm away from the hive entrance and approximately 105 cm from each other (Supplementary Figure S3). The flowers were first placed in front of the boxes, not inside. We tagged the workers that were in the colony with individually numbered tags. On Day 8, we presented a rewarding yellow flower next to one of the boxes during the morning hours (7:00–9:00) and a rewarding blue flower next to the other feeder during the evening hours (16:30–18:30). On Day 10, we moved the flowers into the boxes (Figure 1C,D). First, we left the box lids open for the first hour of reward for each session. After observing five bees feeding inside the boxes, we narrowed down the time of food availability to one hour during the morning and one hour during the evening training sessions. The boxes were non-rewarding during all other times. Given the short distance to the boxes, the need to enter into the box was used to increase the foraging effort and the cost of mistakenly visiting a non-rewarding flower. The whole procedure of tracking and training the colonies took two weeks (Supplementary Figure S1). The times of sunrise and sunset during the entire training period (10 days) were 06:18–06:23 and 18:55–18:43, respectively [32]. We conducted the test day at the end of the second week. During the test day, the flowers were filled with only water. The temperature range was between 25 and 32 °C, and the humidity range was between 52% and 67% [33] on the test day. 

The activity of the control colony was not quantified, but it was qualitatively assessed (none, low, high, etc.).

**Figure 2:**
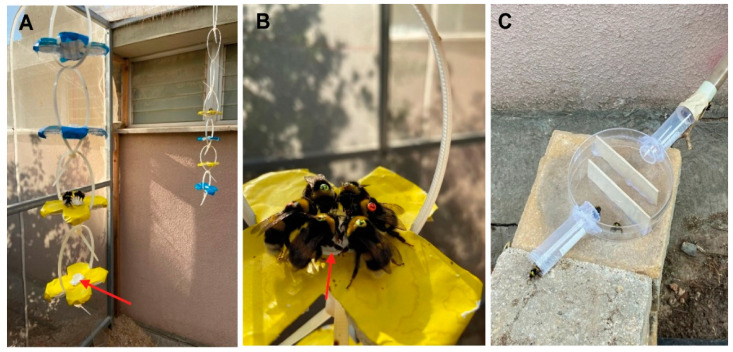
Methodological details of the second experiment. (**A**) Artificial flowers covered with yellow or blue stickers and hung from the ceiling of the flight cage. Each artificial flower was equipped in the middle with a small feeder made from an Eppendorf tube cup (red arrow). (**B**) Tagged bees are seen consuming sugar syrup on a yellow artificial flower. (**C**) The maze at the end of the entrance tube, which was used to slow down bee movement on their way in or out of the nest box.

**Experiment 2.** The overall experimental design (Supplementary Figures S2 and S3) was similar to the first experiment, with the following modifications:

*Flower shape:* We slightly modified the artificial flowers in order to increase the number of bees that could be simultaneously trained. We made the flowers with four petal shapes from a Petri dish and covered these plates with yellow or blue stickers instead of attaching a flower-shaped paper under a Petri dish cover as in the first experiment (Figure 2A,B). Using stickers also helped reduce light reflection, which was occasionally a problem with the Petri dish cover. We replaced the stickers with fresh ones every 2–3 days. The syrup container was a cap of an Eppendorf centrifuge tube (1.5 mL) that was glued to the plate and filled with up to 300 µL commercial sugar syrup.

*Cage learning:* The cage adjustment period lasted two days. At the end of this period, we removed the sugar syrup feeder from the nest. 

*Flower presentation:* On Day 3, we started to present the flowers filled with commercial sugar syrup next to the entrance tube. After that, we hung the artificial flowers using plastic cables hanging from the ceiling of the flight cage. We attracted the first few bees by allowing them to feed on a syrup-coated Pasteur pipette. We slowly transferred them from the entrance to the hanging flowers on the other side of the cage while they were feeding on the Pasteur pipette. We removed the pipette gently right after the bees moved to feed on the flower. This strategy facilitated moving the trained bees from the hive entrance toward the artificial flowers and accelerated the initial training. 

*The influence of body size and worker age/experience on time learning:* The larger sample size in this experiment enabled us to start assessing the influence of body size and age/experience on time learning (see details below). 

On Day 5, in which we recorded five trained bees feeding on the artificial flowers, we transferred the yellow and blue artificial flowers to different corners of the flight cage (same locations as detailed for Exp. 1; Figure 2A). We tagged the workers with green numbered tags (Figure 2B). On Days 6 and 7, the artificial flowers were hung at the two corners of the flight cage with sugar syrup during the daytime, allowing the bees to have enough time to learn to feed on them without restricting the timing of reward. On Day 8, we narrowed the period during which the flowers were rewarding to two hours in the morning (7:00–9:00) and two hours in the evening (16:30–18:30). On Day 11, we further narrowed the times during which the flowers were rewarding to one hour for the yellow flowers during the morning (7:30–8:30) and one hour for the blue flowers during the evening (17:30–18:30). On the same day, we also increased the number of flowers to four of each color such that more bees could obtain a reward. We changed the positions of the colored flowers randomly over the day to make sure the bees learned to associate reward with the flower’s color rather than with its position in the flight cage. On Day 15, we installed a maze at the end of the entrance tube in order to slow down the bees while exiting and entering the nest and to facilitate recording the tag details (Figure 2C). One week before the test day, we tagged all recently emerged untagged bees with orange numbered tags. Thus, worker bees were tagged with two different colors corresponding to their age cohort (total number of tagged = 109, green-tagged = 86, orange-tagged = 23, untagged = 43). On the test day, green-tagged bees were about one week older than orange-tagged bees. The times of sunrise and sunset during the entire training period were 05:37–05:47 and 19:48–19:43, respectively [34].

On the test day, we filled the feeders with tap water (300 µL) in each flower and refilled them at the beginning of each session. We recorded all visitations to the artificial flowers during the following sessions: morning (6:30–9:30), mid-day (11:00–11:30, 13:00–13:30, 15:00–15:30), and evening (16:30–19:30). We recorded the first choice for each bee in each 30-minute interval during each observation session, as well as all visits to the artificial flowers. A visit was defined as a bee landing (all legs on the flower) with her proboscis extended out. Hovering above the flower was not counted as a visit. We also counted how many bees were exiting through the nest box entrance tube per 30 min during these observation sessions in both the focal and control colonies. 

Three observers recorded bee activity during the observation sessions on the test day. Two of them recorded visits to flowers (four flowers each) and the third recorded the number of bees departing from the freely foraging control colony. We automatically video recorded the number of bees exiting the focal colony (connected to the flight cage) using an iPhone 11 camera, which we placed on top of the maze at the end of the entrance tube. Foraging duration in the flight cage was calculated as the difference between the time of departure and the time of return for each trip by a tagged bee. 

The temperature ranged between 26 and 33 °C and the humidity ranged between 44% and 65% during the test day [35].

We froze (−20 °C) the entire colony on the day following the test day. We measured the length of the marginal cell of the forewings (one measurement per bee) as an index of body size (as in [36]).

**Learning Score.** We calculated a Learning Score (LS) for each bee as an index of learning performance. The LS value range from 1.0 to −1.0 and represents the preference of the bee to visit the colored flower that was rewarding during the training sessions at each given time of day. The LS was calculated using the following equation:

$$\mathrm{Learning}\;\mathrm{Score}\;\left(\mathrm{LS}\right)=\left(\mathrm{Nc}-\mathrm{Nw}\right)/\mathrm{Ntotal}$$

Nc: Number of visits to the rewarding colored flowers (correct choice) 

Nw: Number of visits to the non-rewarding colored flowers (wrong choice) 

Ntotal: Total number of visits during the session (Ntotal = Nc + Nw). 

The LS score varies between 1 = only correct choices, to −1 = only wrong choices. An LS of 0 indicates equal number of correct and incorrect choices. We focused on four time intervals during the test day: “pre-morning”—the observations before the time of the morning training (6:30–7:30), “morning”—the time of the morning training (7:30–8:30), “pre-evening”—the observations before the time of the evening training (16:30–17:30), and “evening”—the time of the evening training (17:30–18:30). For the pre-morning and pre-evening sessions, we assumed that the bees anticipated the time of reward availability and therefore the correct choice was the colored flower that was rewarding during the following session.

### 2.3. Statistical Analysis

We used Pearson’s Chi-Square test to test if the frequency of visits and the first choice made by the bee per observation session differed from 50%. We applied the Bonferroni correction for multiple tests. 

We tested if the LS in each time session differed from zero using the one-sample Wilcoxon signed-rank test. We used the Shapiro–Wilk normality test or Kolmogorov–Smirnov normality test to determine if the data distribution was normal. The frequency distribution of bee activity at the colony entrance of the two colonies in Experiment 2 was compared using a two-sample Kolmogorov–Smirnov test. These analyses were performed in R v. 4.0.320 (R Foundation for Statistical Computing, Vienna, Austria). We used 2-way ANOVA to compare the LS and foraging durations of the two age groups in Exp. 2. Two-way ANOVA was performed and all the graphs were created using GraphPad Prism version 5.0 (GraphPad Software, Boston, MA, USA).

For each experiment, we also fit a generalized linear model (GLMM) with a Poisson error structure to test for differences in the number of visits to flowers with different colors (yellow vs. blue) during morning and evening rewarding hours as well as the interaction between color choice and time of day. To account for potential differences among individuals, we included individual ID number as a random variable (random intercept) in both models [37]. We ran all models in R-4.2.1 (R Foundation for Statistical Computing, Vienna, Austria) using the function *glmer* from the package lme4 [38].

## 3. Results

### 3.1. Experiment 1

**Figure 3:**
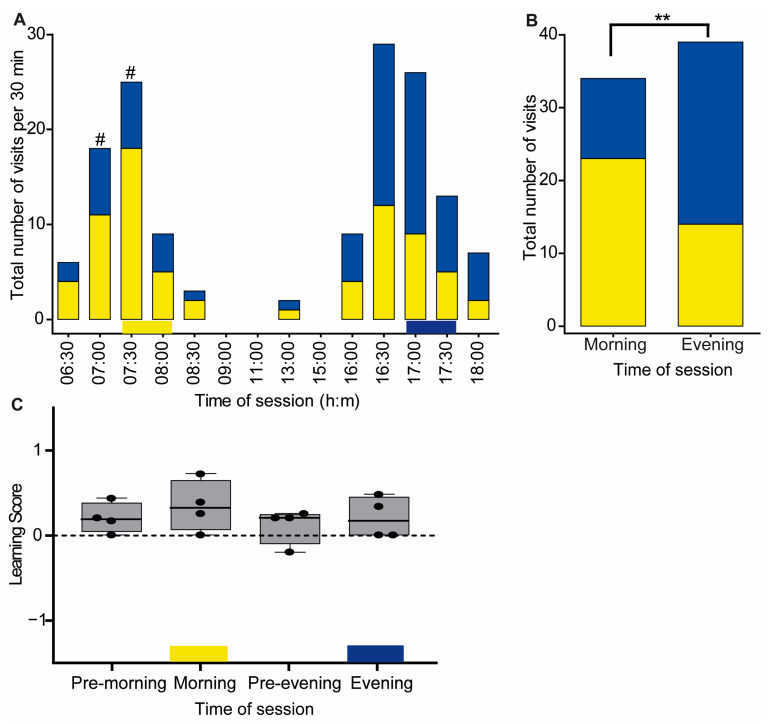
Time–color learning in Experiment 1. (**A**) The total number of visits of trained bees (*n* = 4) to the yellow or blue flower boxes as a function of time of day. The values depict the total number of visits in each half-hour interval. The yellow and blue horizontal bars at the bottom depict the rewarding period and flower color during the training session. #, the probability of visiting the yellow and blue flowers differed in a χ^2^ test (*p* ≤ 0.05) but was not significant after Bonferroni correction. (**B**) A complementary analysis for pooled samples of all visitations during the times in which a reward was provided during the training (7:30–8:30 and 17:00–18:00). Asterisks denote statistical significance for Pearson’s Chi-squared independence test, ** *p* = 0.006. (**C**) Learning Score. The line in the boxplot shows the median and the box frame spans over the first to third quartiles. Each dot shows the LS value of a single bee. The learning score values were not statistically different from zero (one-sample Wilcoxon signed-rank test, *p* > 0.05). Results are shown for visits during one-hour intervals, before the morning training (pre-morning), during the morning training (morning), before the evening training (pre-evening), and during the evening training (evening).

A total of 6 bees out of 80 workers that were tagged in the colony explored the cage during the test day. We limited our analyses to only four of the bees that were recorded visiting rewarding flowers during at least two training days and during both training sessions in each day, and they were therefore assumed to be trained. We used the term “training session” to refer to the time of reward during the training period. The trained bees started visiting the flowers at around 06:30, which was earlier than the time of the morning training session (Figure 3A). Visitation rates increased until around 08:00, and the number of visits decreased towards 09:00, during which time no bee was recorded in the flight cage. Only a single focal bee flew in the cage during the midday observations, but she did not visit the flowers. The foraging activity increased again at around 16:00, just before the evening training session, and reached its peak between 16:30 and 17:30. The last bee returned to the hive at around 18:30. Qualitative assessment of flight out of the control colony also revealed relatively high activity during midday hours, during which time there was almost no activity in the flight cage. 

The bees visited the yellow flower more often during the time corresponding to the morning training session (visits to yellow = 23, blue = 11) and the blue flower more often during the time corresponding to the evening training session (visits to blue = 34, yellow = 21). The frequency of visiting the two colored flowers did not differ from 50%:50% after applying the Bonferroni correction for multiple comparisons (Figure 3A, Table S1). We also did not find a significant difference in the number of visits to flowers as a function of color or time of day with individual ID numbers in the GLMM analysis (likelihood-ratio test: χ^2^ = 8.49; df = 6; *p* = 0.21).

When we limited our analysis to the times of the training sessions, we found that the likelihood of visiting a yellow or blue flower differed between the morning and evening training sessions (Pearson’s Chi-squared independence test, χ^2^ = 7.3255, df = 1, *p*-value = 0.006, Figure 3B). In this analysis, there were also more visits to the yellow flower during the morning training session (Chi-squared test, *p*-value = 0.039); however, visits to the blue flower did not differ from 50%:50% during the evening training session (Chi-squared test, *p*-value = 0.078).

The learning score values (LS, see Section 2) were positive for all of the sessions but did not differ from zero for any of the sessions (Wilcoxon signed-rank exact test: *p*-value > 0.05 for all four time points, Figure 3C). 

### 3.2. Experiment 2

**Figure 4:**
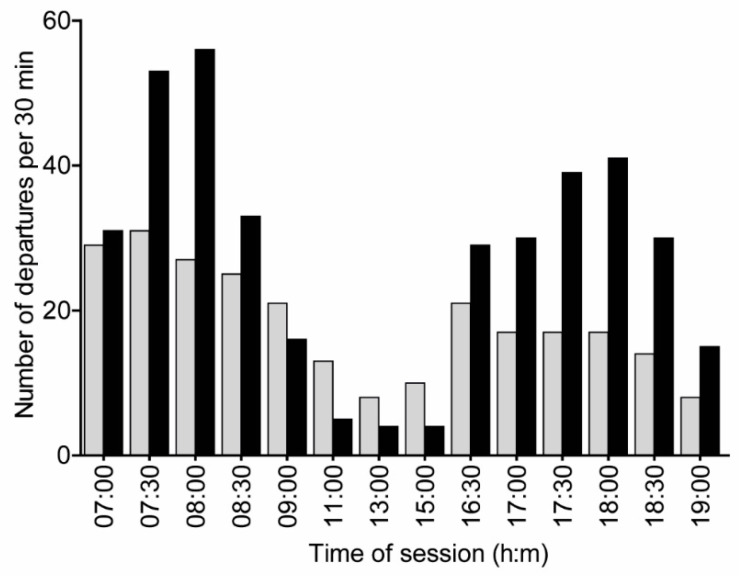
Departures over time during the test day in Experiment 2. The total number of departures was recorded at the entrance tube for the trained colony that was limited to foraging in the flight cage (black bars), and the freely foraging control colony (gray bars). Departures at 6:30 were not recorded for the focal colony. The frequency distributions of activity did not differ between the two colonies but they show a clear trend (two-sample Kolmogorov–Smirnov test; D = 0.5, *p* = 0.06). The focal colony shows higher activity than the control colony during the times of the training sessions and lower activity from 11:00 to 15:00.

Seventeen bees were successfully trained (i.e., they were recorded visiting rewarding flowers during both the morning and evening sessions on at least two training days). Bees started departing from both the focal and control colonies early in the morning of the test day (departures at 6:30 were not recorded for the focal colony, but we know that bees were active during this time because they visited the artificial flowers). Foragers of the control colony, which were freely foraging, were active throughout the day with a 62% drop in activity around noon (11:00–15:30, 8 total departures) relative to the activity at 9:00–9:30 (21 total departures). The total foraging activity in the focal colony (all foragers, not only the trained ones), in which bees were limited to foraging in the flight cage, showed a 75% drop (from 16 departures to 4 departures) in activity level during these hours and the bees were specifically active at and around the times of sugar syrup reward during the training sessions. However, the frequency distributions of activity over time for the two colonies were not statistically significantly different (two-sample Kolmogorov–Smirnov test, D = 0.5, *p*-value = 0.06; Figure 4).

**Figure 5:**
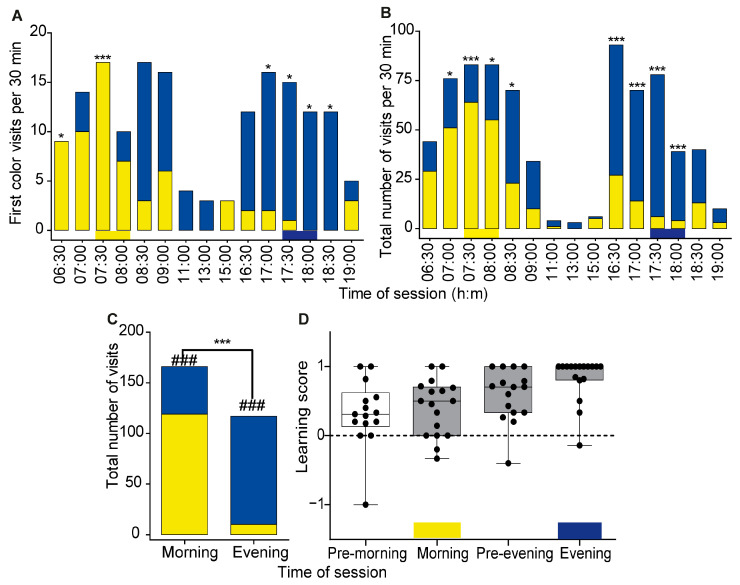
Time–color learning in Experiment 2. (**A**) The first color visited by 17 trained bees. The choice was recorded for each 30-minute observation session. The yellow and blue horizontal bars at the bottom depict the rewarding period and flower color during the training session. (**B**) Total number of visits to the yellow and blue flowers. Asterisks in A and B denote the probability of visiting the yellow and blue flowers differed in a χ^2^ test: * *p* ≤ 0.05, *** *p* ≤ 0.001 after applying the Bonferroni correction. (**C**) A complementary analysis for pooled samples of all visitations during the times in which a reward was provided during the training sessions (7:30–8:30 and 17:30–18:30). Pearson’s Chi-Squared independence test, *** *p* ≤ 0.001. ###, the probability of visiting the yellow or blue flowers differed in a χ^2^ test (*p* ≤ 0.01). (**D**) Learning score (LS) during the test day. A gray box indicates that the difference between the LS and 0 is statistically significant (one-sample Wilcoxon signed-rank test, *p* < 0.05). Other details as in Figure 3C.

The trained bees were more likely to first visit the correct color during the four 30-minute intervals corresponding to the times of the training sessions, and the probability of selecting the correct color was statistically different from a random occurrence in 3 of the 4 intervals (morning: 7:30–8:30; evening: 17:30–18:30) of the training sessions (marked with asterisks in Figure 5A). The preference for yellow was apparent already one hour before the time of the morning training session (6:30–7:00 = 100%, 7:00–7:30 = 71.4%, 7:30–8:00 = 100%). Right after the morning training session, the bees changed their preference and their first visit was switched to the blue flowers. During the evening training session, the bees preferred the blue flowers over the yellow flowers (Table S2). 

A preference for the color of the rewarding flower during the times corresponding to the training sessions was also apparent when we compared the total number of visits to the yellow and blue flowers during the test day. The trained bees visited the yellow flowers more often than the blue flowers during the morning session and in the preceding hour (Figure 5B). After the time corresponding to the morning training session, the bees switched to preferring to visit the blue flowers. The trained bees showed a clear preference for the blue color during the time corresponding to the evening training session. The *p*-values for each time interval are summarized in Table S3. We found significant differences in the bees’ visiting patterns to flowers in the GLMM analysis accounting for individual ID numbers (likelihood-ratio test: χ2 = 68.86; df = 6; *p* < 0.01). While bees significantly increased the number of visits to yellow flowers during the morning rewarding time in comparison to the evening rewarding time, the number of visits to blue flowers increased from the morning to evening rewarding time (Est. 2.22 ± 0.32 SE; CIlow = 1.59; CIup = 2.85; *p* = 0.002).

In a complementary analysis, we analyzed only the visits during the times corresponding to the rewarding sessions. In this analysis, we found that the likelihood of visiting a yellow or blue flower differed between the morning and evening training sessions (Pearson’s Chi-squared independence test, χ^2^ = 110.3, df = 1, *p* < 2.2 × 10^−16^, Figure 5C). Both the total number of visits to the yellow flowers during the morning training session and to the blue flowers during the evening training session differed from 50%:50% (Chi-squared test, *p* < 2.2 × 10^−8^, *p*-value < 3.0 × 10^−19^, respectively).The learning score values (LS) of the trained bees were positive and different from zero during both the morning (median = 0.5, Wilcoxon signed rank test with Bonferroni correction, *p* = 0.02) and evening (median = 1.0, *p* = 0.002) training sessions (Figure 5D). In spite of the notable individual variability, the LS values during the morning and evening training sessions of individual bees were not correlated (Spearman rank correlation analysis; r = −0.03, *p* = 0.91). The LS analysis was consistent with anticipation given that the LS values were positive already one hour before the time of reward, although the LS values were significantly different from 0 only for the period before the evening training session (pre-morning, median LS = 0.31, *p* = 0.17; pre-evening, median learning score LS = 0.7, *p* = 0.01). The LS values of individual bees were not correlated with the number of training days during the training session, but there was a positive trend (Supplementary Figure S4, linear regression, r^2^ = 0.15, *p* = 0.12).

**Figure 6:**
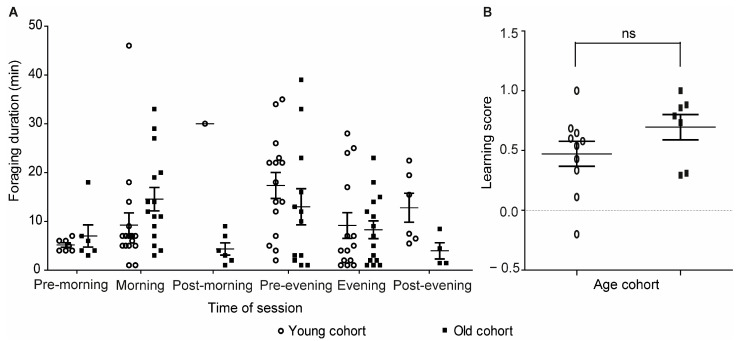
The influence of cohort age on foraging duration and learning performance. (**A**) Foraging duration during the test day. The time sessions were pooled for the observations before the morning training (pre-morning), during the morning training (morning), after the morning training (post-morning), before the evening training (pre-evening), during the evening training (evening), and after the evening training (post-evening). Both time of day, cohort age, and their interaction affected foraging duration (2-way ANOVA; age—*p* = 0.01, time—*p* = 0.01, interaction—*p* = 0.02). (**B**) Learning Scores during the times of the rewarding sessions. Performance during the morning and evening training sessions was pooled together. ns, not significant in an unpaired *t*-test (*p* = 0.16).

Given that we trained bees of two age cohorts in this experiment, we started testing if age or foraging experience influenced learning performance. The training data of the two cohorts are summarized in Table S4. We found that both cohort age and time of day affected the foraging duration of the trained bees, with a significant interaction between these two factors (two-way ANOVA; age, *p* = 0.01, F = 5.62; time of day, *p* = 0.01, F = 2.87; interaction, *p* = 0.02, F = 2.65; Figure 6A). The interaction suggested that the influence of time of day on foraging duration was different for the two age cohorts; bees of the older cohort showed longer foraging duration compared to bees of the younger cohort during morning hours, and an opposite trend was seen during the evening hours. There was no effect of cohort age on learning performance in a test in which we focused only on the times of the morning and evening training sessions (unpaired *t-*test, *p* = 0.16, Figure 6B). 

## 4. Discussion

It is commonly assumed that time-memory is functionally significant. However, the generality of this behavioral capacity is difficult to assess because time-memory has only been studied in a limited number of species. We investigated time-memory in bumble bees, which typically forage over a shorter time and distance than the better studied honey bees [13,14,24,25]. Taken together, our observations showed that the bumble bees learned the time of day and associated it with reward and color, lending credence to the hypothesis that they have time-learning capability. 

First, the trained bees chose to land first on the colored flower that was rewarding during this time of day during the training period (yellow during the morning, blue during the evening). The preference for the learned color was apparent already before the time of reward, which is consistent with anticipation (Figure 5A). Second, the total number of visits to the yellow and blue flowers differed with time of day, with a clear preference for yellow during the morning session and for blue during the evening session. In this analysis, the observed pattern is also consistent with anticipation (Figure 3 and Figure 5). Studies on time-memory in honey bees show that trained bees typically arrive at the feeder before the time of reward with greater anticipation for late-day food resources [39,40,41]. Third, in the two experiments, bees showed a positive learning scores during the rewarding sessions and the values were statistically different from zero in Exp. 2. These findings showed that they learned to choose the colored flowers that were rewarding over the non-rewarding colored flowers based on the time of day (Figure 3C and Figure 5D). Here too, the pattern was consistent with anticipation because their LS values were positive already during the session just before the time of reward during both the morning and evening sessions. The evidence that trained bumble bees anticipate the time of reward is important because anticipation is characteristic of clock-regulated behavior. The LS calculations in Exp. 2 were also consistent with more robust anticipation during the period preceding the evening training session compared to the morning training session (comparison of “pre-morning” and “pre-evening” in Figure 5D). Fourth, in both experiments, the bees showed a clear switch from yellow to blue right after the time of the morning training session. Given that there was no reward during the test day, this observation indicated that their internal clock guided them to switch from seeking reward from yellow to blue flowers during this time. The clear evidence for anticipation and lack of preference during the initial stages of the training suggest that our finding cannot be explained by innate preference for yellow in the morning and blue in the afternoon hours. The rapid switch to blue flowers right after the time of the morning rewarding hours may be explained by an alternative hypothesis that the bumble bees used sequence learning [42,43]. However, given that the yellow flowers were not rewarding during the test day, the motivation to switch to blue flowers cannot be explained without invoking consultation with an internal circadian clock. The possible importance of sequence learning should be further tested in experiments in which time and sequence learning are experimentally uncoupled. 

It is worth mentioning that despite many differences (source colony, sample size, season, shape of artificial flowers, time of sunrise and sunset, temperature throughout the day, humidity) between the two experiments, the results still showed an overall similar pattern that was consistent with the hypothesis that bumble bees can associate time of day with sugar syrup reward and color. The pattern of overall foraging activity may also be seen as consistent with the notion that the trained bees increased their overall activity at times during which they expected to find rewarding flowers in the flight cage. The mid-day decline in foraging activity in Exp. 2 appeared to be higher in the focal colony than in the freely foraging control colony. Although the difference between the two colonies was not statistically significant (*p* = 0.06), the trend was consistent with the premise that the time training affected the temporal organization of foraging activity in the focal colony (Figure 4). Indeed, in previous time training studies with honey bees, the foraging activity of the focal foraging group was typically higher during rewarding times, and little activity was seen during times in which there was no reward during the training sessions [11,40,44,45].

Our observations revealed individual variations among sister bees from the same colonies in both time learning performance and flight duration. To start exploring factors that may contribute to this individual variability, we compared the performance of bees differing in body size and age cohort. There was not sufficient size variability in our sample of foragers to allow rigorous testing of the influence of body size on learning performance. The low variability was probably also influenced by the association between body size and task performance in bumble bees, as larger bees are more likely to forage [36,46,47,48,49]. Additional studies with larger sample sizes and body size variability are needed in order to determine if the body size of the bee influences her performance in time-memory tasks. Our comparisons of bees from two age cohorts did not find an effect on the LS, which was consistent with some previous studies. For example, age and body size did not affect associative odor learning tested by proboscis extension conditioning in *Bombus occidentalis* [50]. Similarly, age and body size had no effect on ecologically relevant foraging performance and associative learning in British *Bombus terrestris* [51]. We did find, however, an effect of age cohort on foraging duration with a significant interaction with time of day. These findings suggest that chronological age, experience, or both influence the daily organization of foraging activity. Additional studies that are beyond the scope of the current study are needed in order to understand the functional significance of these observations. Our small flight cage did not allow rigorous checking of time-place memory, which needs to be tested in field studies or in larger flight cages.

It is commonly accepted that time-memory is functionally significant because it allows animals to visit food patches at times of maximum reward while minimizing the duration of risky foraging (reviewed in [52]). The cost of arriving at the wrong time is specifically high for species such as honey bees that forage over long distances and time duration. Thus, it is reasonable to assume that there is a selective advantage for honey bees to precisely time their visits to flowers (reviewed in [52,53]). It was not clear whether similar strong selection pressures shaped time-memory in species that typically forage over shorter distances and with less sophisticated recruitment system. Our evidence supporting the hypothesis that bumble bees associate time of day with reward and other stimuli shows that efficient time-memory is not limited to species such as honey bees, which evolved sophisticated social foraging over long distances. Our study also adds to the relatively modest list of animals for which there is convincing empirical evidence for time-memory [15,16,17,18,19,20]. Similar comparative studies on species with diverse life histories are necessary in order to understand the generality and adaptive significance of this complex clock-regulated behavior.

## Data Availability

All data are provided in the text, the Supplementary Material, or upon request from the authors.

## Supplementary Materials

The following supporting information can be downloaded at: https://www.mdpi.com/article/10.3390/insects14080707/s1, Figure S1: Experimental outline for Experiment 1; Figure S2: Experimental outline for Experiment 2; Figure S3: Schematic aerial view of the flight cage arena used for time-memory experiments. Foragers (shown as bee icons) enter and exit through the entrance maze from the experimental colony to the flight cage with mesh walls and through the tube connecting the control colony to the outside. Artificial flowers were hung at the far corners of the flight cage.; Figure S4: The relationship between the number of training days and learning score for Experiment 2. Each point shows the Learning Score of an individual bee as a function of the number of days during which she was observed visiting rewarding flowers during the entire training period. A linear regression analysis shows a positive trend that was not statistically significant (r^2^ = 0.15, *p* = 0.12).; Table S1: The total number of visits to yellow or blue flowers on the test day of the first experiment. ‘Session’ refers to the period of observation. The ‘Yellow’ and ‘Blue’ columns summarize the total number of visits to artificial flowers marked with these colors. The *p*-value gives the statistical significance value obtained from a Chi-squared test assuming 50% probability of visiting each one of the colored flowers. The ‘p.adj’ value is the *p*-value after Bonferroni correction.; Table S2: Summary of the first flower color choice of bees on the test day of the second experiment. Table details are as in Table S1. %Correct corresponds to the percentage of correct choices out of the total number of choices made by trained bees.; Table S3: The total number of visits to yellow or blue flowers on the test day of the second experiment. Table details are as in Table S2.; Table S4: Training data for the second experiment, including all tagged foragers that visited the flowers. ‘Session count’ summarizes the number of observation sessions (morning or evening) during which a tagged bee visited the correct color at least once. ‘Old cohort’ and ‘Young cohort’ are the ID numbers of bees visiting flowers. Gray-shaded IDs are trained bees identified during the test day.
