# Supplementary material for: Bumble Bees (Bombus terrestris) Use Time-Memory to Associate Reward with Color and Time of Day
Source: Insects. 2023 Aug 14;14(8):707. doi: 10.3390/insects14080707 (PMC10455649; doi:10.3390/insects14080707)
Supplement: Supplementary file 1 [file insects-14-00707-s001.zip › Time-memory_Suppl.pdf]

Bumble bees (*Bombus terrestris*) use time-memory to associate reward with color and time of day

Ozlem Gonulkirmaz-Cancalar[1], Oded Shertzer[1], and Guy Bloch[1, 2, *]

[1]Department of Ecology, Evolution, and Behavior, The Alexander A. Silberman Institute of Life Sciences, The Hebrew University of Jerusalem, Givat-Ram, Jerusalem, Israel,

[2]The Federmann Center for the Study of Rationality, The Hebrew University of Jerusalem, Jerusalem, Israel

[*] To whom all correspondence should be addressed: Guy Bloch, Department of Ecology, Evolution, and Behavior, The Alexander Silberman Institute of Life Sciences, The Hebrew University of Jerusalem, Berman 114, Jerusalem 9190401, Israel; e-mail: guy.bloch@mail.huji.ac.il

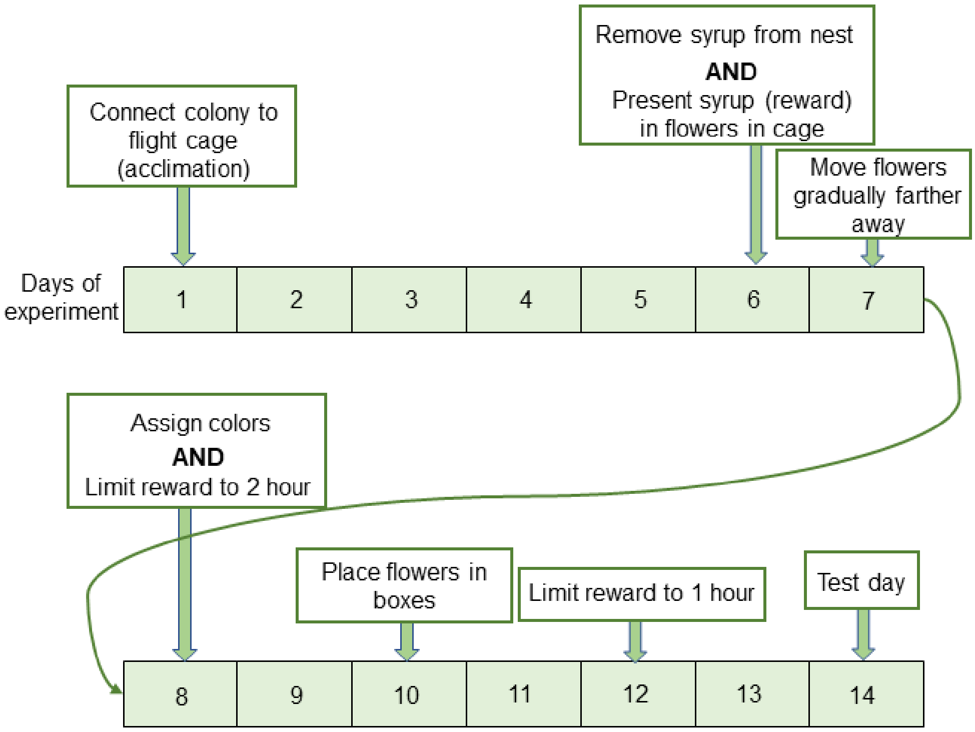

Figure S1. Experimental outline for Experiment 1.

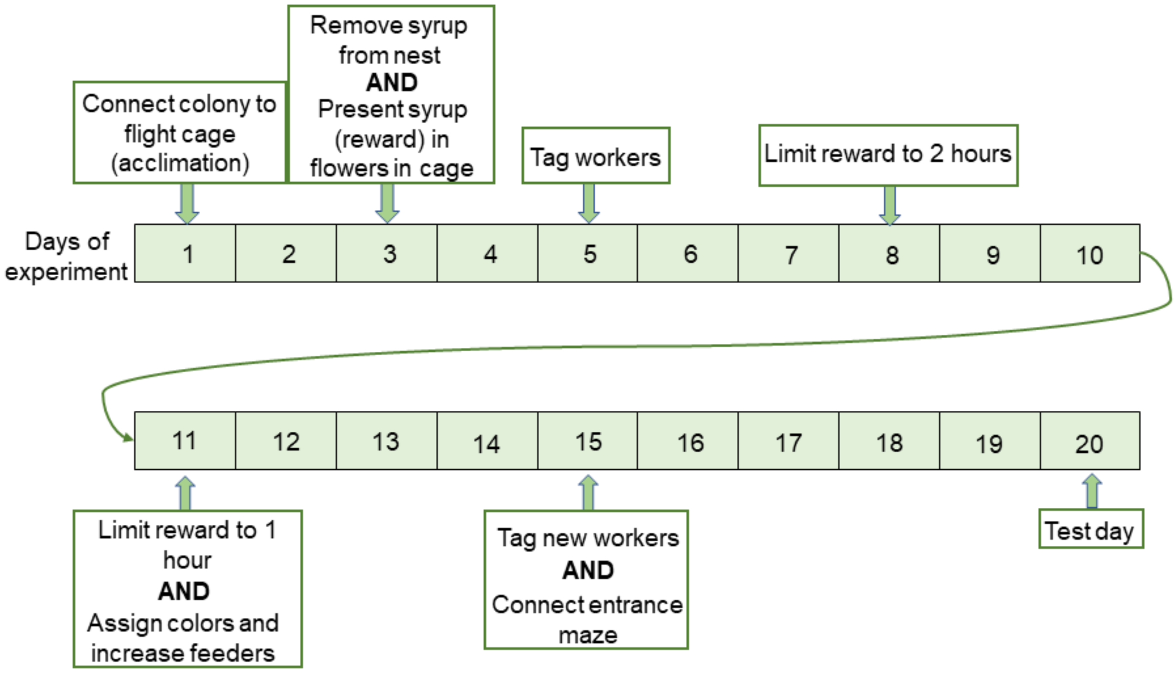

Figure S2. Experimental outline for Experiment 2.

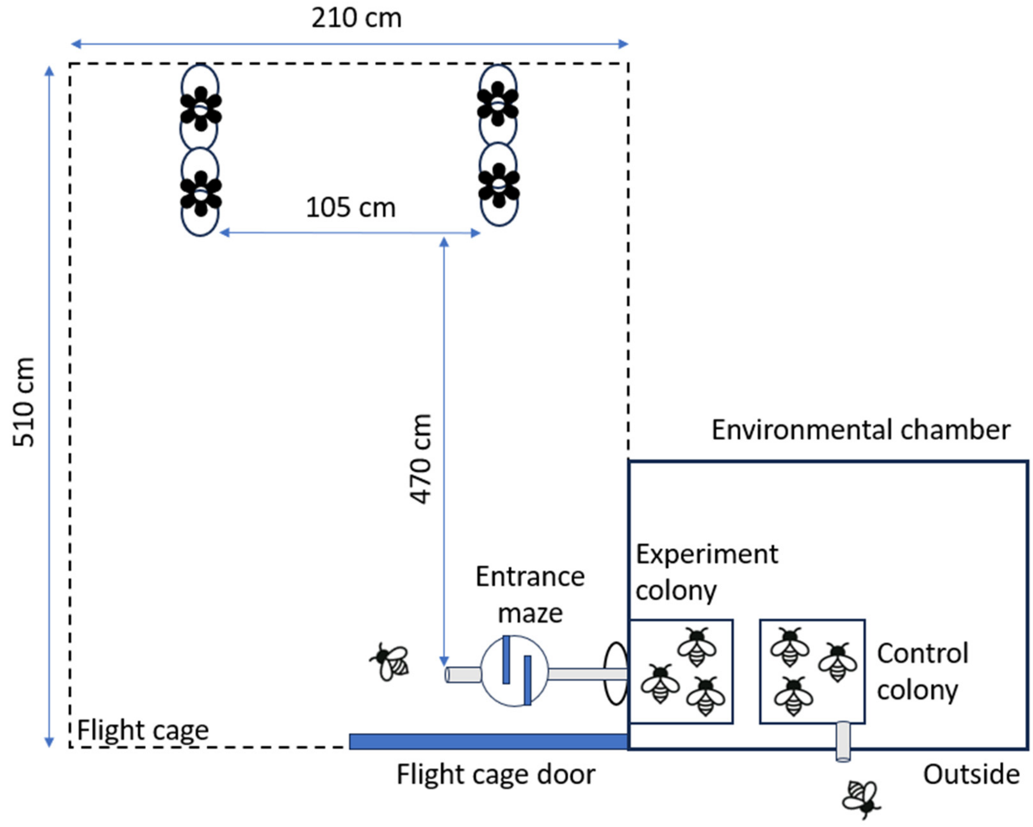

Figure S3. Schematic aerial view of the flight cage arena used for time memory experiments. Foragers (shown as bee icons) go in and out through the entrance maze from the experiment colony to the flight cage with mesh walls and through the tube connecting the control colony to the outside. Artificial flowers were hung at the far-off corners of the flight cage.

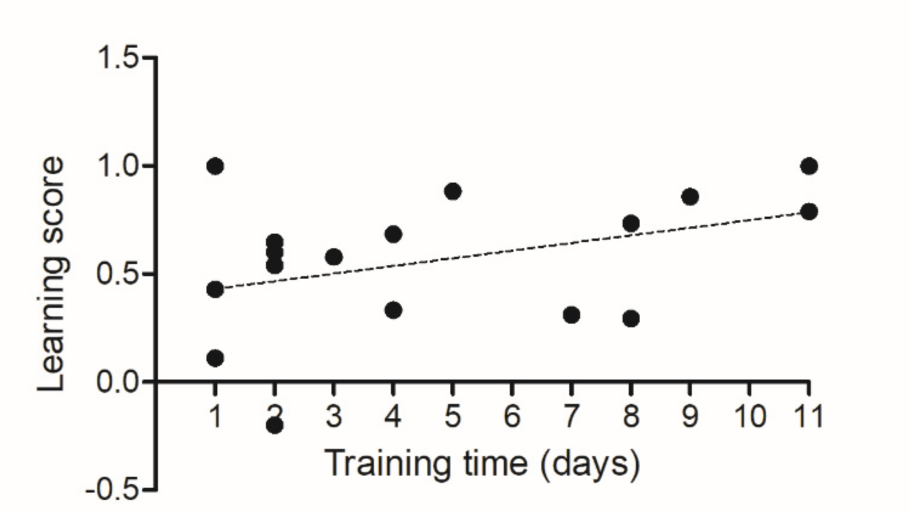

Figure S4. The relationship between the number of training days and the Learning Score in Experiment 2. Each point shows the learning score of an individual bee as a function of the number of days in which she was recorded visiting rewarding flowers during the entire training period. A linear regression analysis shows a positive trend that was not statistically significant ($r^2 = 0.15$, $p = 0.12$).

Table S1 – The total number of visits to 'Yellow' or 'Blue' flowers on the test day of the first experiment. 'Session' refers to the period of observation. The 'Yellow' and 'Blue' columns summarize the total number of visits to artificial flowers marked with these colors. The '*p*.value' gives the statistical significance value obtained from a Chi-squared test assuming 50% to visit each one of the colors. '*p*.adj' values are the p values after Bonferroni correction.

Session	Yellow	Blue	*p*.value	*p*.adj
PreMorning	15	9	0.220	1
Morning	23	11	0.039	0.277
PostMorning	2	1	0.563	1
Midday	1	1	1	1
PreEvening	16	22	0.330	1
Evening	14	25	0.078	0.547
PostEvening	2	5	0.256	1

Table S2 – Summary of the first choice of bees on the test day of the second experiment. Table details are as in Table S1. %Correct corresponds to the percentage of correct choices out of the total number of choices made by trained bees.

Session	Yellow	Blue	% Correct	*p*.value	*p*.adj
06:30	9	0	100	0.002	0.032
07:00	10	4	71.4	0.108	1
07:30	17	0	100	3.74E-05	0.001
08:00	7	3	70	0.205	1
08:30	3	14	17.7	0.007	0.091
09:00	6	10	37.5	0.317	1
16:30	2	10	83.3	0.02	0.251
17:00	2	14	87.5	0.002	0.032
17:30	1	14	93.3	0.001	0.009
18:00	0	12	100	0.001	0.006
18:30	0	12	100	0.001	0.006
19:00	3	2	40	0.654	1

Table S3 – The total number of visits to 'Yellow' or 'Blue' flowers on the test day of the second experiment. Table details are as in Table S2.

Session	Yellow	Blue	% Correct	*p*.value	*p*.adj
06:30	29	15	65.9	0.035	0.417
07:00	51	25	67.1	0.003	0.034
07:30	64	19	77.1	7.83E-07	9.40E-06
08:00	55	28	66.3	0.003	0.036
08:30	23	47	32.9	0.004	0.049
09:00	10	24	29.4	0.016	0.196
16:30	27	66	71.0	5.25E-05	6.30E-04
17:00	14	56	80.0	5.16E-07	6.20E-06
17:30	6	72	92.3	7.83E-14	9.40E-13
18:00	4	35	89.7	6.90E-07	8.28E-06
18:30	13	27	67.5	0.026	0.322
19:00	3	7	70.0	0.205	1.000000E+00

Table S4 – Training data for the second experiment. That table includes data for all tagged foragers that visited the flowers. ‘Session count’ summarizes the number of observation sessions (morning or evening) in which a tagged bee visited the correct color at least once. The numbers inside the ‘Old cohort’ and ‘Young cohort’ columns are ID codes of individually tagged bees that visited flowers. Gray-shaded IDs are trained bees identified during the test day.

Old cohort	Session count	Young cohort	Session count
8	9	12	2
11	6	18	2
14	1	42	1
17	1	52	1
22	17	54	2
28	14	58	6
35	15	64	1
37	19	65	1
38	3	73	2
42	1	79	4
46	8	81	3
51	1	83	8
53	12	87	4
58	12	89	5
60	10	96	1
72	1	97	2
74	15		
79	2		
80	1		
84	2		
86	15		
